# Baseline Drug Treatments as Indicators of Increased Risk of COVID-19 Mortality in Spain and Italy

**DOI:** 10.3390/ijerph182211786

**Published:** 2021-11-10

**Authors:** Kevin Bliek-Bueno, Sara Mucherino, Beatriz Poblador-Plou, Francisca González-Rubio, Mercedes Aza-Pascual-Salcedo, Valentina Orlando, Mercedes Clerencia-Sierra, Ignatios Ioakeim-Skoufa, Enrico Coscioni, Jonás Carmona-Pírez, Alessandro Perrella, Ugo Trama, Alexandra Prados-Torres, Enrica Menditto, Antonio Gimeno-Miguel

**Affiliations:** 1 EpiChron Research Group, Aragon Health Sciences Institute (IACS), IIS Aragón, Miguel Servet University Hospital, 50009 Zaragoza, Spain; kbliek@salud.aragon.es (K.B.-B.); bpoblador.iacs@aragon.es (B.P.-P.); franciscagonzalezrubio@gmail.com (F.G.-R.); maza@salud.aragon.es (M.A.-P.-S.); mclerencia@salud.aragon.es (M.C.-S.); Ignatios.IoakeimSkoufa@fhi.no (I.I.-S.); jcarmona@iisaragon.es (J.C.-P.); sprados.iacs@aragon.es (A.P.-T.); 2 Teaching Unit of Preventive Medicine and Public Health, Miguel Servet University Hospital, 50009 Zaragoza, Spain; 3 Centro Interdipartimentale di Ricerca in Farmacoeconomia e Farmacoutilizzazione (CIRFF), Center of Drug Utilization and Pharmacoeconomics, Department of Pharmacy, University of Naples Federico II, 80131 Naples, Italy; valentina.orlando@unina.it (V.O.); enrica.menditto@unina.it (E.M.); 4 Health Services Research on Chronic Patients Network (REDISSEC), Institute of Health Carlos III (ISCIII), 28222 Madrid, Spain; 5 Delicias-Sur Primary Care Health Centre, Aragon Health Service (SALUD), 50009 Zaragoza, Spain; 6 Drug Utilization Work Group, Spanish Society of Family and Community Medicine (semFYC), 08009 Barcelona, Spain; 7 Primary Care Pharmacy Service Zaragoza III, Aragon Health Service (SALUD), 50017 Zaragoza, Spain; 8 Aragon Health Service (SALUD), Miguel Servet University Hospital, 50009 Zaragoza, Spain; 9 WHO Collaborating Centre for Drug Statistics Methodology, Norwegian Institute of Public Health, 0213 Oslo, Norway; 10 Department of Drug Statistics, Division of Health Data and Digitalisation, Norwegian Institute of Public Health, 0213 Oslo, Norway; 11 Division of Cardiac Surgery, AOU San Giovanni di Dio e Ruggi d’Aragona, 84131 Salerno, Italy; enrico.coscioni@regione.campania.it; 12 Infectious Disease of Healthcare Direction, AORN Antonio Cardarelli, 80131 Naples, Italy; alessandro.perrella@aocardarelli.it; 13 Regional Pharmaceutical Unit, Campania Region, 80143 Naples, Italy; ugo.trama@regione.campania.it

**Keywords:** COVID-19, medications, drugs, mortality, real-world data

## Abstract

This study aims to identify baseline medications that, as a proxy for the diseases they are dispensed for, are associated with increased risk of mortality in COVID-19 patients from two regions in Spain and Italy using real-world data. We conducted a cross-country, retrospective, observational study including 8570 individuals from both regions with confirmed SARS-CoV-2 infection between 4 March and 17 April 2020, and followed them for a minimum of 30 days to allow sufficient time for the studied event, in this case death, to occur. Baseline demographic variables and all drugs dispensed in community pharmacies three months prior to infection were extracted from the PRECOVID Study cohort (Aragon, Spain) and the Campania Region Database (Campania, Italy) and analyzed using logistic regression models. Results show that the presence at baseline of potassium-sparing agents, antipsychotics, vasodilators, high-ceiling diuretics, antithrombotic agents, vitamin B12, folic acid, and antiepileptics were systematically associated with mortality in COVID-19 patients from both countries. Treatments for chronic cardiovascular and metabolic diseases, systemic inflammation, and processes with increased risk of thrombosis as proxies for the conditions they are intended for can serve as timely indicators of an increased likelihood of mortality after the infection, and the assessment of pharmacological profiles can be an additional approach to the identification of at-risk individuals in clinical practice.

## 1. Introduction

Around 182 million COVID-19 cases, including almost 4 million deaths, have been reported to the World Health Organization (WHO) worldwide since the beginning of the pandemic in 2020 [1]. The wide range of clinical manifestations and possible outcomes of the infection, from asymptomatic to critical or even deadly, has made identifying underlying conditions that affect COVID-19 mortality a top priority.

Age is widely considered the most influential factor in infection mortality [2,3]. Others include male sex, and cardiovascular and metabolic comorbidities such as diabetes, obesity, chronic renal failure and chronic heart disease [4]. A fundamental aspect common to these conditions and COVID-19 pathophysiology is its repercussion on the body’s inflammatory response and immune and coagulation systems [5].

Given their critical role on health outcomes, population-based studies using pharmacological information have set their focus on heavily age-related factors such as anticholinergic risk and polypharmacy, mainly based on anticholinergic scales or the number of prescribed or dispensed drugs [6]. However, and apart from the effects of the drugs themselves on COVID-19 prognosis, the use of dispensation data prior to the infection can be an additional tool for risk stratification in infected patients, especially when information on the underlying comorbidities is unavailable or when the pressure on the healthcare system demands expeditious risk evaluation and decision making. Though less common, studying medication profiles as a proxy of disease and their association with COVID-19 severity can be a swift and objective approach to the characterization of vulnerable patient profiles for the improvement of their clinical management and the development of targeted prevention strategies for at-risk individuals [7,8,9].

The objective of this study was to identify baseline medications associated with an increased risk of mortality that can serve as timely indicators of severity in COVID-19 patients from two regions in Spain and Italy, two of the hardest hit countries during the initial SARS-CoV-2 outbreak in Europe.

## 2. Materials and Methods

### 2.1. Design and Study Population

We conducted a cross-country, retrospective observational study using real-world data. Our study population was obtained from two cohorts comprising users of the public health services of two European regions: the PRECOVID Study cohort in Aragon (Spain) and the Campania Region Database (CaReDB) in Campania (Italy). From this point on, the cohorts will be referred to simply as Aragon and Campania.

For this study, we included all the individuals from both regions with confirmed SARS-CoV-2 infection between 4 March 2020 and 17 April 2020 (enrollment period). We followed patients for a minimum of 30 days from the date of inclusion in the cohort to allow sufficient time for the studied event, in this case death, to occur. Follow-up was stopped invariably on 17 May 2020.

### 2.2. Variables and Data Sources

The following baseline variables were analyzed for each individual at the date of entry: sex, age (0–14, 15–44, 45–64, 65–79, ≥80 years), and all drugs dispensed in community pharmacies during the three months prior to infection. Drugs were coded using the Anatomical Therapeutic Chemical (ATC) classification system at its third level (i.e., pharmacological subgroup). To facilitate clinical interpretation, we used the Rx-Risk Index, a validated comorbidity index that maps the dispensed ATC pharmacological and therapeutic groups to their corresponding comorbidity categories [10]. Patient data were obtained from healthcare databases with routinely collected information, which are detailed below.

The CaReDB includes demographic information on the approximately 6 million residents living in Campania (roughly 10% of the Italian population) and links with their pharmaceutical dispensation electronic records. Data in CaReDB were validated in previous drug utilization studies [11,12,13]. COVID-19 information in the region was collected via a specific surveillance system, which was developed during the initial stages of the pandemic to detect all cases identified by reverse transcription-polymerase chain reaction (RT-PCR). The diagnostic algorithm was based on the protocol released by the WHO and consisted of the collection of nasopharyngeal swab samples tested with at least two real-time RT-PCT assays targeting different genes of SARS-CoV-2 [14]. A unique identifier, later encrypted for anonymization purposes, served to connect all the aforementioned data sources. The governance board of Unità del Farmaco della Regione Campania granted permission to the researchers of the Centro di Ricerca in Farmacoeconomia e Farmacoutilizzazione (CIRFF) to use anonymized data for this study.

The PRECOVID Study cohort in Aragon included all individuals with laboratory-confirmed infection by SARS-CoV-2 in the region (reference population, 1.3 million inhabitants). The regional public health service provides free-of-charge healthcare to approximately 95% of the population of Aragon. Patient data were obtained from the Aragon Health System linking, at a patient level and in a pseudo-anonymized form, the information contained in the health system users’ database, primary care and hospital pharmaceutical billing records, and an ad hoc registry implemented for COVID-19 surveillance in the region. The protocol of this study was approved by the Clinical Research Ethics Committee of Aragon (CEICA; protocol number PI20/226). CEICA waived the requirement to obtain informed consent from patients given the epidemiological nature of the project with anonymized data. The research protocol adheres to the tenets of the Declaration of Helsinki 1975 and its later amendments.

### 2.3. Statistical Analysis

Demographic and drug utilization patterns of all patients who tested positive for SARS-CoV-2 (alive and deceased at the end of follow-up) were reported as means and standard deviations or as absolute and relative frequencies and proportions, stratified by sex.

Logistic regression models were performed to analyze the association between the presence of each drug dispensed at baseline and the likelihood of mortality during the 30-day follow-up period in each region, stratified by sex. Age-adjusted odds ratios (ORs) were calculated together with their respective 95% confidence intervals (CI). Only medications with an overall prevalence equal to or greater than 1% were considered for the analysis in order to avoid spurious findings. Statistical significance was set at *p* < 0.05.

Data management was performed with Microsoft SQL server (version 2018). All analyses were performed in Stata software (Version 12.0, StataCorp LLC, College Station, TX, USA), SPSS software for Windows (version 17.1, SPSS Inc., Chicago, IL, USA) and platform R (version 3.6, The R Formulation for Statistical Computing, Vienna, Austria).

## 3. Results

A total of 8570 individuals were included in this study (Table 1). Of these, 4412 belonged to Aragon (58.8% women, mean age of 67.7 (standard deviation, SD, 20.7)) and 4158 (44.3% women, mean age 63.0 (SD 17.9)) to Campania. 771 (47.2% women, mean age of 84.2 (SD 10.0)) and 455 (35.4% women, mean age 74.3 (SD 13.5)) individuals died during follow-up in Aragon and Campania, respectively. Three in four deceased patients were 80 years of age and over in Aragon, as opposed to Campania where only one in three were above that threshold. Men died at an earlier age than women in both regions. The mean number of dispensed medications was higher in deceased individuals than in those alive by the end of follow-up in both regions (7.6 vs. 4.0 drugs in Aragon and 6.6 vs. 3.0 in Campania) (Table 2).

Drug families with the highest dispensation prevalence during the three months prior to COVID-19 infection in both regions were (Aragon, Campania) drugs for peptic ulcer and gastro-esophageal reflux disease (GORD) (35.3%, 35.1%), antithrombotic agents (22.8%, 24.2%), beta-blocking agents (11.7%, 16.4%), high-ceiling diuretics (14.4%, 6.3%), and blood-glucose-lowering drugs (11.2%, 7.7%) (Table 3). Drug dispensation was higher in Aragon than in Campania in all pharmacological groups except for antithrombotic agents and drugs for peptic ulcer and GORD, with notable differences in antidepressants (21.2%, 5.6%) and antipsychotics (10.9%, 3.6%).

The following drug families were significantly associated with 30-day mortality in patients with COVID-19 in both regions (OR Aragon, OR Campania): potassium-sparing agents (1.98, 3.01), antipsychotics (1.92, 3.01), vasodilators used in cardiac diseases (1.76, 3.23), high-ceiling diuretics (1.87, 2.54), antithrombotic agents (1.58, 2.54), vitamin B12, folic acid (1.59, 2.70), and antiepileptics (1.61, 2.72).

The dispensation of some drug families increased the risk of mortality in COVID-19 patients in only one of the two regions (OR (95% CI)), or data for them were unavailable at the time. Angiotensin-converting enzyme inhibitors (ACE) (2.25 (1.69–3.00)), lipid-modifying agents (2.06 (1.63–2.59)), peripherally acting antiadrenergic agents (1.88 (1.10–3.21)) and corticosteroids for systemic use (1.58 (1.23–2.02)), among others, were associated with a higher risk of mortality in Campania, while anxiolytics (1.41 (1.15–1.72)), immunosuppressants (1.89 (1.01–3.54)), and anti-infectives (1.95 (1.03–3.69)) showed increased risk only in Aragon. These results are presented as Supporting Information (see Supplementary Table S1).

## 4. Discussion

In this comparative study we identified the main pharmacological groups that, as a proxy for the baseline comorbidities they are dispensed for, were associated with an increased risk of mortality in COVID-19 patients from two regions in Spain and Italy, evidencing common results. We found that the dispensation of drugs related to the treatment of chronic cardiovascular and metabolic diseases such as β-blocking agents, vasodilators, antithrombotic agents, diuretics, and blood glucose-lowering drugs dispensed three months prior to infection were associated with higher mortality risks in these patients. Dispensed vitamin B12 and folic acid supplementations, often linked to deficits during systemic inflammation and processes with increased risk of thrombosis, were also found to increase the risk of mortality after the infection.

The first weeks of the pandemic already underscored the now notorious effects of advanced age on the risk of COVID-19-related mortality [15]. Almost three in four deceased individuals in Aragon, a region where residential care homes for the elderly were hit hardest during the first months of the pandemic, were 80 years of age or older, while in Campania, a region with one of the youngest populations in Italy, this proportion was much lower, approximately one in three. Chronic diseases and multimorbidity, which are more frequent in older patients, as well as immune-senescence, are some of the added factors that could contribute to the higher morbidity and mortality risks in these age groups after COVID-19 infection [16].

Different methodological approaches (for example, in the selection of chronic diseases and data sources), settings (in-hospital and out-hospital patients, special treatment during admission, healthcare system’s capacity), and regions (sociodemographic characteristics, varying social-distancing measures and health policies) have been used for the characterization of the population at risk of poor prognosis. These discrepancies cumbered the comparability and interpretation of the different findings, especially during the first stages of the pandemic. Despite these limitations, certain underlying conditions were identified as risk factors of adverse outcomes in COVID-19 patients across the board; amongst them diabetes, obesity, chronic cardiovascular diseases, and chronic renal failure [17]. Consequently, pharmacological treatments as proxies for these chronic conditions can also be expected to be associated with a higher risk of mortality. Our study identified the chronic dispensation at baseline of β-blocking agents, vasodilators used in cardiac diseases, antithrombotic agents, high-ceiling diuretics, potassium-sparing agents, and blood glucose-lowering drugs as indicators of increased risk in both regions of Spain and Italy. Though previous studies have shown that the treatments themselves are safe during COVID-19 infection and should not be discontinued, real-world data on their dispensation can be an objective and prompt tool to detect patients at risk and anticipate severe outcomes [18,19,20].

According to our findings, the predominant pharmacological profile of a high-risk individual in Aragon was that of a geriatric patient with heart failure and dysrhythmias, while in Campania, it was a hypertensive patient with cardiometabolic diseases. The influence of pharmacological profiles on the clinical evolution of COVID-19 patients may be significant for both decision-making and prognosis. Many patients, especially the elderly, have multimorbidity and are in treatment with several drugs (polypharmacy) and thus are susceptible to potentially inappropriate medication, drug-drug and drug-disease interactions, adverse drug reactions, and therapeutic cascades [21]. Swift identification of disease patterns with a higher risk of severe outcomes, both directly or through the medications used to treat them, is vital when designing prevention strategies in the changing landscape of the pandemic, especially considering the pathophysiological characteristics of the infection remain unclear and considering new drugs can be developed or implemented in future guidelines.

In addition to cardiovascular and cardiometabolic medication, we found associations between other drug categories and COVID-19 mortality. The use of antigout preparations could be linked to treatments with antidiuretics and heart failure, which are also associated with a higher risk of negative outcomes [22]. Vitamin B12 and folic acid supplementation are prescribed in situations of vitamin B12 deficiency, and were the only drugs that could not be mapped to a corresponding comorbidity category using the Rx-Risk Index. However, it has been well established that elevated homocysteine levels during vitamin B12 deficiency are associated with higher cardiovascular risk, and could be related to antithrombotic treatment implementation in these patients [23]. Systemic inflammation and conditions with an increased risk of thrombosis are important risk factors of adverse outcomes in infected patients [5]. One of the most critical findings in our study was the identification of antidepressants, antipsychotics, and antiepileptics as high-risk medications of COVID-19 mortality in both Aragon and Campania. The use of these drugs with high anticholinergic activity seems to be higher in Aragon, including anxiolytics, probably due to the older mean age of the population, but the role of these drug groups and the conditions they are indicative of still require further research.

The core strength of this study is its population-based approach. We used real-world data to include all confirmed COVID-19 cases and all the drugs dispensed during the three months prior to infection in the regions of Campania and Aragon. Another strength is its comparative nature, allowing the contrasting of results from two settings with unique characteristics from two of the countries that suffered the most during the initial stages of the pandemic in Europe. Its main limitation is that it only included drugs dispensed from community pharmacies under prescription, and not over-the-counter medications or in-hospital treatments. Another limitation is that we lacked the information on the chronic diagnoses that justified the dispensation of each drug. Though we mapped each pharmacological code to the corresponding comorbidity group based on their most frequent indications, some drugs can be used for a wider number of pathologies. Furthermore, some of the information for certain drug groups of interest, such as hypnotics, sedatives, and anxiolytics, were only available in one of the two regions and could not be compared. Future studies, including all relevant pharmacological groups and their combinations, are called for to better understand their role as potential mortality risk indicators in COVID-19 patients.

## 5. Conclusions

Results show that baseline chronic treatments for known COVID-19 risk factors such as chronic cardiovascular and metabolic diseases, systemic inflammation, and processes with increased risk of thrombosis, are systematically associated with mortality in infected patients. When chronic diagnoses are unavailable or complex, the assessment of pharmacological profiles as proxies can be an alternative approach to a swift identification of at-risk individuals in clinical practice.

## Figures and Tables

**Table 1:** Demographic characteristics of the study population, by region and follow-up status (i.e., exitus no/yes).

Region	Aragon, Spain						Campania, Italy					
Sex	All (N = 4412)		Women (N = 2593)		Men (N = 1819)		All (N = 4158)		Women (N = 1843)		Men (N = 2315)	
Exitus	No (N = 3641)	Yes (N = 771)	No (N = 2229)	Yes (N = 364)	No (N = 1412)	Yes (N = 407)	No (N = 3703)	Yes (N = 455)	No (N = 1682)	Yes (N = 161)	No (N = 2021)	Yes (N = 294)
Age (mean, sd)	61.8 (19.5)	84.2 (10.0)	60.8 (20.2)	87.2 (8.2)	63.3 (18.1)	81.5 (10.7)	60.7 (17.8)	74.3 (13.5)	61.8 (18.8)	76.2 (14.9)	59.8 (16.8)	73.0 (12.3)
Age (*n*, %)												
≤14	6 (0.16)	0 (0)	0 (0)	0 (0)	6 (0.42)	0 (0)	156 (4.2)	2 (0.4)	67 (4.0)	1 (1.2)	89 (4.4)	0 (0)
15–44	721 (19.8)	1 (0.13)	516 (23.1)	0 (0)	205 (14.5)	1 (0.25)	1145 (30.9)	45 (9.9)	531 (31.6)	16 (9.9)	614 (30.4)	29 (9.9)
45–64	1384 (38.0)	36 (4.67)	844 (37.9)	7 (1.92)	540 (38.2)	29 (7.13)	1578 (42.6)	106 (23.3)	674 (40.1)	36 (22.4)	904 (44.7)	70 (23.8)
65–79	670 (18.4)	172 (22.3)	318 (14.3)	51 (14.0)	352 (24.9)	121 (29.7)	575 (15.5)	169 (37.1)	257 (15.3)	43 (26.7)	318 (15.7)	126 (42.9)
≥80	860 (23.6)	562 (72.9)	551 (24.7)	306 (84.1)	309 (21.9)	256 (62.9)	249 (6.7)	133 (29.2)	153 (9.1)	64 (39.8)	96 (4.8)	69 (23.5)

**Table 2:** Number of drugs dispensed of the study population, by region and follow-up status (i.e., exitus no/yes).

Region	Aragon, Spain						Campania, Italy					
Sex	All (N = 4412)		Women (N = 2593)		Men (N = 1819)		All (N = 4158)		Women (N = 1843)		Men (N = 2315)	
Exitus	No (N = 3641)	Yes (N = 771)	No (N = 2229)	Yes (N = 364)	No (N = 1412)	Yes (N = 407)	No (N = 3703)	Yes (N = 455)	No (N = 1682)	Yes (N = 161)	No (N = 2021)	Yes (N = 294)
Drugs (mean, sd)	4.0 (3.9)	7.6 (4.2)	4.0 (3.9)	7.6 (4.0)	4.0 (4.0)	7.7 (4.3)	3.0 (2.9)	6.6 (4.2)	3.3 (3.5)	6.8 (5.1)	3.2 (3.6)	6.4 (5.2)

**Table 3:** Drug families in which dispensation within the three months prior to COVID-19 infection was consistently associated with 30-day mortality in patients from both regions studied.

Region		Aragon, Spain				Campania, Italy			
Sex		All	All	Women	Men	All	All	Women	Men
ATC Code	Drug Family	Prev. (%)	OR (95% CI)	OR (95% CI)	OR (95% CI)	Prev. (%)	OR (95% CI)	OR (95% CI)	OR (95% CI)
C03D	Potassium-sparing agents	3.0	1.98 (1.36–2.89)	1.70 (1.03–2.81)	2.32 (1.32–4.07)	2.4	3.01 (1.91–4.77)	2.32 (1.06–5.08)	3.60 (2.01–6.42)
N05A	Antipsychotics	10.9	1.92 (1.55–2.39)	2.05 (1.54–2.74)	1.76 (1.26–2.45)	3.6	3.01 (2.06–4.39)	4.44 (2.76–7.14)	2.16 (1.12–4.18)
C01D	Vasodilators used in cardiac diseases	2.1	1.76 (1.14–2.72)	1.27 (0.66–2.44)	2.30 (1.27–4.16)	1.2	3.23 (1.73–6.04)	3.58 (1.40–9.15)	3.11 (1.34–7.22)
C03C	High-ceiling diuretics	14.4	1.87 (1.53–2.28)	1.65 (1.26–2.16)	2.11 (1.57–2.85)	6.3	2.54 (1.87–3.46)	2.48 (1.53–4.02)	2.71 (1.81–4.05)
M04A	Antigout preparations	5.5	1.48 (1.10–1.99)	2.22 (1.30–3.81)	1.26 (0.88–1.79)	4.6	3.45 (2.48–4.81)	4.86 (2.87–8.24)	2.75 (1.80–4.21)
B01A	Antithrombotic agents	22.8	1.58 (1.32–1.90)	1.60 (1.23–2.07)	1.56 (1.21–2.02)	24.2	2.54 (2.08–3.11)	3.11 (2.24–4.33)	2.25 (1.74–2.91)
B03B	Vitamin B12 and folic acid	7.6	1.59 (1.21–2.04)	1.59 (1.10–2.31)	1.59 (1.11–2.28)	3.6	2.70 (1.83–3.99)	3.13 (1.83–5.35)	2.69 (1.52–4.77)
N03A	Antiepileptics	8.8	1.61 (1.26–2.07)	1.86 (1.33–2.60)	1.37 (0.95–1.99)	4.4	2.72 (1.91–3.88)	3.47 (2.11–5.71)	2.39 (1.44–3.98)
C07A	Beta blocking agents	11.7	1.52 (1.23–1.89)	1.33 (0.98–1.82)	1.70 (1.25–2.30)	16.4	2.28 (1.83–2.85)	2.18 (1.50–3.17)	2.31 (1.75–3.05)
A02B	Drugs for peptic ulcer and GORD	35.3	1.54 (1.28–1.84)	1.28 (0.99–1.67)	1.78 (1.38–2.29)	35.1	2.27 (1.87–2.77)	2.70 (1.94–3.75)	2.11 (1.65–2.70)
G04C	Drugs used in benign prostatic hypertrophy	8.5	n.a.	n.a.	1.10 (0.84–1.45)	6.3	n.a.	n.a.	2.07 (1.50–2.87)
A10B	Blood glucose lowering drugs	11.2	1.27 (1.02–1.59)	1.33 (0.96–1.85)	1.23 (0.91–1.66)	7.7	2.19 (1.64–2.94)	2.48 (1.48–4.15)	1.97 (1.38–2.81)
N06A	Antidepressants	21.2	1.44 (1.20–1.76)	1.44 (1.12–1.86)	1.44 (1.07–1.93)	5.6	2.36 (1.70–3.28)	2.58 (1.65–4.03)	2.79 (1.68–4.63)

ATC: Anatomical-Therapeutic-Chemical code at the third level; Prev.: prevalence; OR: odds ratio; CI: confidence interval; GORD: Gastro-oesophageal reflux disease; n.a.: not applicable. Only drugs with an overall prevalence equal to or greater than 1% and with a statistically significant association with mortality (*p* < 0.05) in both regions are presented. The complete list of drugs from both regions is available as Supplementary Material.

## Data Availability

Data used in this study cannot be publicly shared because of restrictions imposed by the Aragon Health Sciences Institute (IACS) and asserted by the CEICA. Nonetheless, data can be made available for potential collaborations upon reasonable request to the Principal Investigators Alexandra Prados-Torres (sprados.iacs@aragon.es) and Enrica Menditto (enrica.menditto@unina.it).

## Supplementary Materials

The following are available online at https://www.mdpi.com/article/10.3390/ijerph182211786/s1. Table S1: Dispensation rates of the complete list of drugs studied and adjusted odds ratios of 30-day mortality in each region.
